# Posttraumatic growth following a first episode of psychosis: a mixed methods research protocol using a convergent design

**DOI:** 10.1186/s12888-016-0977-4

**Published:** 2016-07-25

**Authors:** Gerald Jordan, Ashok Malla, Srividya N. Iyer

**Affiliations:** 1 Prevention and Early Intervention Program for Psychosis, Douglas Mental Health University Institute, 6875 Boul LaSalle, Verdun, QC H4H 1R3 Canada; 2 Department of Psychiatry, ACCESS Open Minds/Esprits ouverts, Prevention and Early Intervention Program for Psychoses, Douglas Mental Health University Institute; McGill University, 6875 Boulevard LaSalle, Verdun, QC H4H 1R3 Canada; 3 Department of Psychiatry, McGill University, Montreal, QC Canada

**Keywords:** First episode psychosis, Posttraumatic growth, Coping, Social support, Resilience, Recovery, Mixed methods

## Abstract

**Background:**

The suffering people experience following a first episode of psychosis is great, and has been well-investigated. Conversely, potential positive outcomes following a first episode of psychosis have been under-investigated. One such outcome that may result from a first episode of psychosis is posttraumatic growth, or a positive aftermath following the trauma of a first psychotic episode. While posttraumatic growth has been described following other physical and mental illnesses, posttraumatic growth has received very little attention following a first episode of psychosis. To address this research gap, we will conduct a mixed methods study aimed at answering two research questions: 1) How do people experience posttraumatic growth following a first episode of psychosis? 2) What predicts, or facilitates, posttraumatic growth following a first episode of psychosis?

**Methods/design:**

The research questions will be investigated using a mixed methods convergent design. All participants will be service-users being offered treatment for a first episode of psychosis at a specialized early intervention service for young people with psychosis, as well as their case managers.. A qualitative descriptive methodology will guide data-collection through semi-structured interviews with service-users. Service-users and case managers will complete questionnaires related to posttraumatic growth and its potential predictors using quantitative methods. These predictors include the impact a first episode of psychosis on service-users’ lives, the coping strategies they use; the level of social support they enjoy; and their experiences of resilience and recovery. Qualitative data will be subject to thematic analysis, quantitative data will be subject to multiple regression analyses, and results from both methods will be combined to answer the research questions in a holistic way.

**Discussion:**

Findings from this study are expected to show that in addition to suffering, people with a first episode of psychosis may experience positive changes. This study will be one of few to have investigated posttraumatic growth following a first episode of psychosis, and will be the first to do so with a mixed methods approach.

## Background

A first episode of psychosis (FEP), which is typically characterized by the onset of experiences such as hallucinations and delusions, is often a traumatic experience that leaves young people feeling alienated from themselves and the world around them, and often results in disrupted occupational, educational and social trajectories [1–3]. However, as has been documented with various traumatic events including life-threatening physical health problems, war, abuse, natural disasters, death of loved ones, etc., positive changes (such as developing stronger connections with others, or becoming more mature, learning how to better handle stress) can occur alongside and following illness or trauma [4].

Such positive changes as a result of illness have been referred to as posttraumatic growth (PTG). On the one hand, it could be argued that PTG forms part of the recovery process from experiences such as FEP; they have been described by service-users as a component, or process of, recovery [5–11]; and have been theorized by some to be a transformational form of recovery [12]. On the other hand, it could also be argued that PTG represents a state beyond recovery (which can be viewed as a return to normal, or baseline functioning, as was suggested by Carver, [13]) Some have also described PTG as resilience (for reference, see [4]); however, PTG may be separate from the concept of resilience, or evidence of resilience; with positive personal, social, cultural and institutional factors (which may constitute resilience) laying the foundations for PTG. Conversely, growth may also be resilience-enhancing; for instance, an individual who develops stronger relationships with parents may come to have better social support the next time such support is needed.

While PTG has been explored in relatively older individuals with multiple-episodes of psychosis, or more chronic psychotic disorders over several years [14], few studies have examined the phenomenon of PTG in younger people in treatment for a FEP. Studying PTG in a younger population is important because young people are in the stages of forming important educational and occupational trajectories, as well as negotiating identity issues. Having a positive narrative that young people may draw from may inspire hope in those who suffer, and may help young people, their families and treatment provider’s structure care in such a way as to promote PTG.

To date, only two studies which have explored PTG following FEP have used quantitative approaches [5, 15]. Of these, only one examined predictors of PTG (which included recovery, trauma and self-disclosure) [5]. Both were based on very small convenience samples (one study had a sample size of 34, while the other had a sample size of 2; and in the second study, quantitative measures were included as part of a case study not guided by a clear methodology). These methodological issues may limit the generalizability and validity of their findings. Furthermore, only one qualitative study has explored the phenomenon of PTG, and revealed that through FEP, participants developed improved relationships with others; experienced enhanced perspective taking, confirmation of the character of others; a greater appreciation of life; new possibilities; and a stronger sense of self. However, the authors did not explore what participants felt facilitated PTG [16].

PTG-like processes have been described in other qualitative studies; however, the aim of these studies was not to discover PTG in participants, but rather to elucidate other aspects or processes important for young people who have recently experienced FEP. These include studies on the process of recovery from FEP (in which participants described recovery more in terms of a return to normal, and less so in terms of PTG) [6–11]; the experience of first seeking treatment for FEP [17]; an evaluation of a specialized early intervention service treating FEP [18]; and experiences of what it is like to be a family member of a young person with FEP [19]. Findings from these studies yielded information on how people have grown from FEP, namely, that they appreciated life more, experienced increased spirituality, developed new interests, and strengthened bonds with others. However, the lack of focus on PTG in these qualitative studies may have resulted in a narrower depiction of PTG following FEP than would have resulted from a study focused specifically on PTG.

While independent qualitative and quantitative studies have their own strengths, combining both methods to address research questions may also be beneficial [20]. For instance, including a qualitative component into a study may yield the benefit of producing a richer, more nuanced account of a phenomenon; conversely, applying quantitative methods may yield the benefits of testing models predicting PTG or associations of PTG with possibly inter-connected concepts (e.g., resilience and recovery). Hence, a methodological approach which addresses the shortcomings of studies on PTG conducted so far by including a larger sample of well-characterized persons with psychosis and by leveraging a mixed methods approach may significantly advance our understanding of PTG following FEP.

Such a study of the aspects and facilitators of PTG following FEP may help service-users, their loved ones and treatment teams foster PTG; may help counter the highly stigmatizing views society holds about people who have experienced FEP; and may provide a strong, much needed message of hope about the experience of psychosis.

In summary, the study’s larger aims are to investigate both aspects of PTG following FEP (e.g., better relationships with others, experiencing a stronger self, etc.) and facilitators/predictors of PTG (e.g., coping, social support, etc.), using a mixed methods approach.

### Research questions

Qualitative Research Questions: 1) What are the aspects of PTG service-users experience following a FEP 2) What do service-users perceive as facilitating aspects of PTG following FEP?

Quantitative Research Questions: 1) What aspects of PTG are most frequently endorsed by service-users following FEP? 2) Which factors predict PTG following a FEP?

## Methods/design

The research questions will be investigated using a mixed methods approach [20] so that we can capture objectively the extent to which PTG and its various components are endorsed by persons with FEP and also understand subjectively how PTG is experienced following a FEP. Similarly, while the quantitative measures will allow us to determine the extent to which postulated factors predict PTG, using qualitative methods will yield subjective perceptions of the role played by these factors. Additional aspects (beyond those hypothesized) may also emerge in the qualitative analyses as influencing whether and how individuals experience growth following a FEP, which will be considered.

The project will employ a convergent design [20, 21] that entails conducting separate qualitative and quantitative methods integrated at all steps of the research process.

For this project, quantitative and qualitative methods will be mixed at the level of research questions, data collection, data analysis, and interpretation of results. We will conduct both study methods simultaneously.

Ethical approval for this project was granted by the McGill University Institutional Review Board (which has jurisdiction at both recruitment sites). All eligible participants will be explained the study protocol and those who agree will sign informed consent forms prior to participation.

### Qualitative methods of the study

In-depth, semi-structured, individual interviews lasting approximately an hour will be conducted in either English or French. The aim of the interviews will be to elucidate subjective experiences of PTG and to capture what participants feel enabled them to grow. In addition, we will also explore any negative consequences resulting from FEP, and be open to additional ways participants have grown as well as additional facilitators of PTG, to capture a more nuanced understanding of participants’ experiences. Interviews will be audiotaped and transcribed verbatim. A qualitative descriptive approach will guide all aspects of the qualitative methods we employ to derive a comprehensive description of participants’ experiences that is both deep and meaningful [22].

Throughout the study, the primary investigator will be reflexive and aware of how his perceptions and beliefs intersect at various junctures with the project to actively guide the project. These perceptions and beliefs emerge from having had previous experience working within the context of a disability studies framework which considers “pathology” to be a variation in human functioning which can be celebrated [23], and having had highly positive and rewarding experiences helping individuals with psychotic disorders. In terms of the design of this protocol, an awareness of the disability studies framework helped conceptualize the role that enabling environments play in influencing the extent to which people may be able to achieve PTG.

### Quantitative methods of the study

**Fig. 1:**
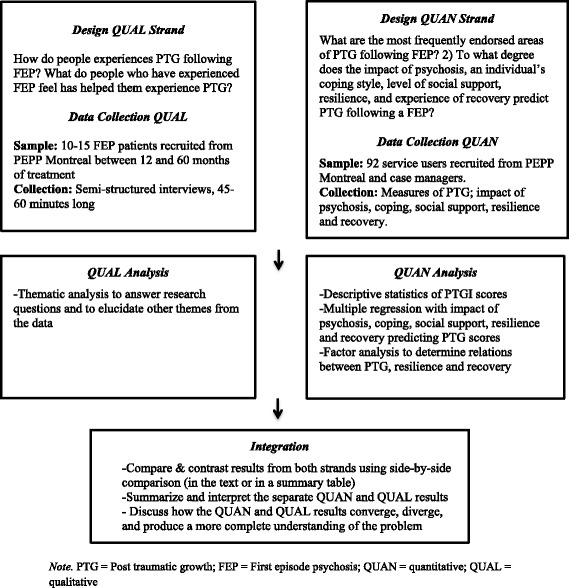
Study design

Questionnaires assessing PTG and five hypothesized predictors of PTG (i.e., the impact that FEP had on service-user’s lives, coping strategies, levels of social support, and experiences of recovery and resilience) will be administered to service-users recruited. In addition, each service-user’s case manager will complete an adapted third-person version of the Posttraumatic Growth Inventory (Fig. 1).

### Recruitment criteria and setting

Participants will include service-users being offered treatment for a FEP and key treatment providers (i.e., their case managers) recruited from two specialized early intervention services for FEP in the McGill University Network - the Prevention and Early Intervention Program for Psychoses (PEPP) at the Douglas Mental Health University Institute and at the McGill University Health Centre. These programs are located in the lower west and central parts of Montreal, Quebec, respectively, treat all potential cases of FEP in their respective catchment area, and together serve a population of over 500,000 people. Individuals are accepted for treatment if they are experiencing a FEP not attributable to substance use or an organic brain condition (e.g., epilepsy); are between the ages of 14 and 35; have not previously taken antipsychotic medication for more than 30 days; have an IQ above 70; and are able to communicate in either English or French. Service-users are treated for between two and five years, during which they are offered close follow-up through intensive case management and antipsychotic medication. In addition to case management, other psychosocial services are provided as needed such as cognitive behavioral therapy for social anxiety [24].

In addition to being followed at PEPP, potential participants must be clinically stable (defined through consensus by psychiatrists and case managers at weekly meetings as not being in a relapse); must have received treatment for a minimum of six months and a maximum of five years; and must be between the ages of 18 and 35. We chose to focus on this age range because of known differences between adolescents and adults on a range of early clinical and functional indicators [25]. Younger individuals also have different needs and are at different developmental junctures compared to older individuals.

### Sampling strategy and power calculations

When employing qualitative methods, we will recruit participants who have experienced some degree of growth following their FEP using a purposive sampling technique (as evaluated by their case manager and/or psychiatrist). A maximum variation strategy will be employed, and we will attempt to ensure that both genders and a range of SES backgrounds are well-represented in our sample, as gender and SES have been shown to influence PTG [26]. We estimate that between 10 and 15 participants will be recruited. Given that this project is being completed within the scope of a doctoral progam, we may not be able to reach the point of theoretical saturation.

When employing quantitative methods, we will recruit all service-users at PEPP who meet study recruitment criteria. In order to achieve 80 % power, a sample size of 92 participants will be needed to obtain a moderate effect size (f^2^ = .15) in a multiple regression analysis (i.e., the main analysis we will perform) with five predictor variables tested at an alpha level of .05. Since no previous study that has examined PTG included the predictors of interest in this study, our power estimations were based on an assumption of a moderate effect size, which is consistent with effect sizes generally observed in behavioral sciences [27], and with meta-analyses examining PTG following non-psychotic illnesses [28]. An 80 % power level was chosen—relative to a higher power level—to decrease the chances of making a type-1 error. Power was calculated using G*Power version 10.

### Measures and procedures

#### Qualitative interview guide

A semi-structured interview guide developed by the primary investigator will be used to conduct in-depth interviews. The interview guide was validated through feedback from service-users and their family members, as well as case managers, psychiatrists, and research evaluators at PEPP. Each stakeholder group made suggestions and modifications to the content of the guide.

The guide contains open-ended questions to help the interviewer probe why participants felt they sought help at PEPP; the ways which those experiences led to changes to the self, relationships with others, new life possibilities, appreciation for life, and spirituality. The guide will also help ascertain what participants feel has facilitated PTG, and will probe for whether or not participant’s thought coping, social support, experiencing recovery or other aspects of resilience (such as one’s connection to their cultural practices) helped them achieve PTG. Probes will be phrased in an open-ended format, and will help the us determine the subjective experience of PTG following FEP and facilitators of PTG to ensure that mixing at the level of data collection can occur. However, we will remain open to participants discussing areas of growth or facilitators of growth outside what is being specifically assessed in the guide. Also, the interview guide is expected to evolve following each interview, leading to modifications in the guide. Summary notes of interviews will also be produced following each interview, which will include notes related to the research questions; aspects of the interview which the interviewer found interesting or challenging and worthy of reflection; as well as the ease at which the interviewee spoke, and seemed comfortable describing their experiences.

#### Quantitative measures

Decisions on which measures to use were based on existing literature on factors important for the development of PTG following physical illnesses [4, 28–30] and input from consultations with clinician-scientists and clinicians at PEPP. Thus far, studies have shown that experiences perceived as mildly or severely negative do not foster growth, while experiences perceived as moderately negative do [4]. Other important factors predictive of PTG following physical illnesses include having adaptive strategies to cope with adversity as well as people to draw on in times of need [29]. Being recovered may also be important [6–11]. Being resilient, defined by some as the ability to bounce back from stressful situations, has also been found to be important [29, 31].

The included measures are well-established, have previously been used in psychosis populations, and are well-validated and reliable. All questionnaires have been translated into French in line with the World Health Organization’s [32] instructions for translation and adaptation of instruments. Pen-and-paper or online versions in either English or in French will be administered depending on the preference of the participants and modality of completion (pen-and-paper vs. online) will be noted.

### Posttraumatic growth

PTG will be measured using the Posttraumatic Growth Inventory [33], which is the most widely used, validated scale to measure PTG following traumatic illness or events [30]. The PTG Inventory is a 21-item scale which assesses growth in five domains, namely: relating to others (e.g., having compassion for others), developing new possibilities (e.g., I established a new path in life), personal strength (e.g., knowing I can handle difficulties), spiritual change (e.g., I have a stronger religious faith), and appreciation for life (e.g., my priorities about what is important in life). As done in prior research examining outsider perspectives of PTG [34], the questionnaire has been adapted to the third person for case managers to fill out with reference to the PTG experienced by their clients (e.g. “my client established a new path in life?”).

### Predictors of posttraumatic growth

The impact of psychosis will be measured using the Subjective Experiences of Psychosis Scale, which is a well-validated, service-user generated scale that measures both the positive (e.g., feelings of empowerment) and negative impacts (e.g., feelings of depression) that psychosis can have on an individual’s life. Individuals will be asked to separately rate the positive and negative impact of psychosis on 31 items using a 5-point Likert scale. Participants will be classified as being mildly, moderately or very negatively impacted by FEP by separating average ratings on the negative impact subscale into three discrete categories representing being mildly, moderately and greatly affected.

Coping will be measured using the Brief COPE scale [35] which is a validated, 21-item measure of coping strategies abbreviated from the original scale [36]. The scale measures 14 different coping strategies, namely, active coping (e.g., I’ve been taking action to try to make the situation better); planning (e.g., I’ve been trying to come up with a strategy about what to do); positive reframing (e.g., I’ve been looking for something good in what is happening); acceptance (e.g., I’ve been learning to live with it); humor (e.g., I’ve been making jokes about it); religion (e.g., I’ve been praying or meditating); emotional support (e.g., I’ve been getting emotional support from others); instrumental support (e.g., I’ve been getting help and advice from other people); self-distraction (e.g., I’ve been turning to work or other activities to take my mind off things); denial (e.g., I’ve been refusing to believe it has happened); venting (e.g., I’ve been expressing my negative feelings); substance use (e.g., I’ve been using alcohol or other drugs to make myself feel better); behavioral disengagement (e.g., I’ve been giving up the attempt to cope); and self-blame (e.g., I’ve been criticizing myself).

Social support will be measured with the Multidimensional Scale of Perceived Social Support [37, 38], which is a validated, 12-item measure of support received from significant others (e.g., there is a special person who is around when I am in need), family (e.g., my family really tries to help me), and friends (e.g., I have friends with whom I can share my joys and sorrows).

Recovery will be measured using the Recovery Assessment Scale [39], a validated measure of perceptions of recovery following the experience of psychosis. This 41-item (each rated on a 5-point Likert scale) scale measures recovery in 5 domains, namely, personal confidence and hope (e.g., fear doesn’t stop me from living the way I want to), willingness to ask for help (e.g., I know when to ask for help), goal and success orientation (e.g., I have my own plan for how to stay or become well), reliance on others (e.g., even when I don’t believe in myself, other people do) and lack of domination by symptoms (e.g., my symptoms interfere less and less with my life).

Since most valid and reliable measures of resilience are specific to developmental periods (either childhood/youth or adulthood) that do not completely overlap with the age range of the sample to be recruited (i.e., 18–35), two measures of resilience will be used. When measuring resilience in service-users between the ages of 18 and 23 we will use the Child and Youth Resilience Measure—Youth version [40], across 8 domains; namely, personal skills (e.g., I try to finish what I start); peer support (e.g., I think my friends care about me when times are hard); social skills (e.g., I know where to go in my community to get help); caregiver support (e.g., I feel my parents/caregivers know a lot about me); spiritual life (spiritual beliefs are a source of strength for me); education (e.g., I feel I belong at school); and connection with culture (e.g., I like the way my community celebrates things). When measuring resilience in service-users over the age of 23, the adult version of the Child and Youth Resilience Measure will be used [40], which measures resilience in the same domains as the child and youth version. Both versions are fairly similar. We will conduct factor analyses of data from the resilience measure filled out by persons below and above 23 years and retain resilience data for those above 23 only if it yields factor scores comparable to those among persons with FEP below the age of 23 and to those in the original factor analysis of the scales.

### Covariates

Other potential variables will be considered on the basis on their correlations with PTG and if they are described as important during the qualitative interviews. If additional covariates are considered, more participants will be recruited to satisfy power requirements. Potential covariates include demographic factors (e.g., age, gender, level of education, time since FEP), symptomatology (e.g., measured using the Scale for the Assessment of Positive Symptoms [41] and Scale for the Assessment of Negative Symptoms [42]); functioning (e.g., using the Strauss Carpenter Scale [43, 44]; premorbid adjustment (e.g., using the Premorbid Adjustment Scale [45]); and medication adherence (using self-reports, and clinical notes [46]). These measures are administered at regular intervals throughout follow-up as part of the standard PEPP evaluation protocol [24].

### Data analyses

Data analyses will occur in three ways. Separate qualitative and qualitative analyses will be conducted followed by a merging of results from both methods.

### Qualitative data analyses

The primary investigator will conduct all interviews in English or French depending on service-user preferences. Interviews will be audiotaped and transcribed verbatim (transcripts will included all words, hesitations, laughter and background noise) by an outsourced company and checked for accuracy by at least two researchers. A thematic analysis of transcripts will be conducted, using the inductive and deductive procedure outlined by Braun and Clarke [47] in order to develop themes related to our research questions. In addition to developing themes to answer the research questions, we will also develop themes outside this scope which may arise through the co-construction of meaning between participants and the analyst; for instance, we may attempt to tease apart differences in how service-users describe their experience of PTG from how they describe recovery, resilience, and facilitators of growth more broadly. The process of coding and building themes using a deductive approach will be informed by literature on PTG.

An inductive and deductive approach to coding will be applied to form initial codes, which will undergo further refinement and additional coding until themes are developed. Two individuals will engage in the coding process and the building of theme. Using multiple coders will help enrich the analysis. Detailed memos will be kept by both coders, which will assist with the interpretative process of the analysis. A thematic map of the interrelationships between themes will be also produced.

The methods described by Braun and Clarke will be used to ensure adequate rigour. These include ensuring that data are transcribed accurately and verified by two individuals; paying attention to each data item; coding thoroughly to ensure the coherence, consistency and distinctiveness of themes and their correspondence with the data; conducting interpretive, rather than superficial, analysis; ensuring a match between extracts and analysis, that the analysis presents a narrative, and that a balance between narrative and extracts exists; giving ourselves time to properly analyze the data; and ensuring the fidelity of the final written report. Finally, we will take steps to ensure that we pay adequate attention to contextual factors shaping the experience of or factors contributing to PTG. Such factors may include the organizational climate of services, and the degree to which they are (or are not) open, empowering and resilience enhancing; the degree to which participants feel their environments and systems outside of the treatment settings are resilience-enhancing and accessible to them; as well as factors at play within the Quebec healthcare system, which may revolve around the recent re-organization of the healthcare system.

### Quantitative data analyses

Descriptive statistics will be computed based on the PTG Inventory scores, separately for service-users and case managers. Correlations between any potential covariates and PTG Inventory scores will be computed. A multiple stepwise regression will be conducted to determine predictors of PTG, with potential covariates in the first block, followed by the five hypothesized predictor variables in the second block and PTG as the outcome variable. A factor analysis of items of the PTGI, Child and Youth Resilience Measure, and the Recovery Assessment Scale will also be conducted to distinguish the statistical differences between these constructs.

### Mixed method data analysis

Results generated from the qualitative and quantitative methods used will be compared after results from each method are analyzed separately to form an overall interpretation of the experience of PTG following FEP and the factors that facilitate PTG. Convergence (i.e., similar results from the qualitative and quantitative methods used), and divergence (i.e., contradictory or different results from the qualitative and quantitative methods used) will be examined using two side-by-side comparison tables related to each research question (see Table 1).

**Table 1:** An example of a mixed methods results table

Theme	Qualitative	Quantitative
	Themes related to Subjective Experiences of Posttraumatic Growth According to Importance	Endorsement of Domains related to Subjective Experience of Posttraumatic Growth According to Magnitude
T1	Developing Positive Character Traits	Greater Appreciation for Life
T2	Positive Lifestyle Changes	Developing Positive Character Traits
T3	Stronger Connections with Family	Stronger Connections with Family
T4	Stronger Connections with Friends	Stronger Connections with Friends
T5	Integration of Experience	Positive Lifestyle Changes
T6	Greater Appreciation for Life	Greater Religiosity
T7	Greater Religiosity	
T8^a^	Becoming more authentic	
T9^a^	Greater Civic Engagement	
	Themes related to Subjective Experience of Facilitators of Posttraumatic Growth According to Importance	Predictors of Posttraumatic Growth According to Magnitude of Standardized Beta Coefficients
T1	Coping	Recovery
T2	Social Support	Coping
T3^a^	Medication	Social Support
T4^a^	Symptom Remission	Resilience
T5	Socio Economic Status	Impact of Psychosis
T6	Recovery	
T7	Resilience	
T8	Impact of Psychosis	

*Note*: ^a^ = Themes which may arise spontaneously

In the first table, themes related to PTG following FEP generated from the qualitative analyses, and the means and standard deviations of each subscale of the PTG Inventory derived from quantitative analyses, will be entered in separate columns and ordered according to importance (for themes) or magnitude (based on means and standard deviations). In the second table, factors perceived as important for the development of PTG derived from the qualitative analyses, and significant predictors of PTG derived from the quantitative analyses along with their beta weights, will be displayed in separate columns and ordered according to importance and magnitude, respectively. Convergence and divergence will be interpreted by examining these tables. Specifically, themes which were developed from results using qualitative methods which do not match what was measured using quantitative methods will be discussed and interpreted in terms of how they complement and enrich our understanding of PTG following FEP. An overall picture of convergence and divergence will be presented in the results section, and interpreted in the discussion section.

## Discussion

The aim of the proposed study is to understand PTG following FEP, and what psychosocial factors are important for its development. This study is among three studies [5, 15, 16] to *directly* explore PTG following FEP since many of studies reporting PTG, or PTG-like processes, have been embedded in studies with aims other than discovering PTG [6–11, 17–19]. We expect the findings from this study to have greater validity, and generate a better understanding,  because of the application of a mixed-methods convergent design. Using qualitative methods, our study will capture subjective experiences of PTG that cannot be appreciated using exclusively quantitative approaches. Using quantitative methods, our study will help document the extent to which PTG is experienced in FEP and will help establish a predictive model of factors influencing PTG.

Multiple stakeholders (e.g., service-users, family members and clinicians) have pointed out that the current narrative around FEP can seem disempowering and biased towards the suffering and negative impacts of psychosis. Researchers have also opined that the positive sequelae and growth resulting from adversity are worthy of investigation [48]. Findings from this study may therefore give greater hope to service-users with psychosis, and inform hope-inspiring, strengths-based treatment approaches to facilitate positive changes among service-users. Since our sample of service-users will be well-characterized and come from a well-defined catchment area, results generated through the quantitative methods of our study are likely to be highly generalizable. Furthermore, we believe the qualitative results will be analytically generalizable by adding depth to the dominant, medical-model conceptualization of FEP and its aftermath, as our findings will describe both the positive and negative aftermath of FEP. To our knowledge, this is one of few studies to directly examine PTG following FEP using mixed methods as part of its overall methodology, which may be important given that PTG has not been systematically examined previously in FEP.

However, this study will not be without its limitations. The PTG inventory has not been specifically validated for use in FEP. However, the scale has been successfully used and found to be valid with multiple populations in multiple contexts (varying illnesses, varying geographic regions, etc.). This strengthens the argument for the use of this scale in our study. Further, the use of qualitative methods in our study may help to validate the use of PTG Inventory in FEP or make a case for the creation of a FEP-specific scale for PTG.

Our choice of factors to test as predictors of PTG is based on previous PTG research with individuals who had not experienced a FEP. As such, these factors may not be the most pertinent ones. Service user perceptions of what is important for the development of growth following the experience of a psychosis will be elicited by this project, which will address this limitation and suggest further avenues of research.

While we believe our study is a needed step in understanding the experience of PTG following FEP, we believe that future studies should explore how other people in service-users’ social network’s also grow through their loved one’s FEP; and how treatment providers themselves may also grow, through providing services to those in need. Such investigations would demonstrate how varying stakeholder groups may benefit from the negative experiences of FEP. Finally, instead of relying on case managers to identify service-users who have experienced PTG, identifying participants who have experienced PTG from their responses to the PTG Inventory for subsequent interviews may result in a greater participant pool for interviews. However, this recruitment process seemed difficult to carry out because this work is being conducted as a Doctoral project.

## Abbreviations

FEP, first episode psychosis; PTG, posttraumatic growth
